# Why 2D layout in 3D images matters: evidence from visual search and eyetracking

**DOI:** 10.16910/jemr.16.1.4

**Published:** 2023-03-31

**Authors:** Linda Krauze, Mara Delesa-Velina, Tatjana Pladere, Gunta Krumina

**Affiliations:** University of Latvia, Riga, Latvia

**Keywords:** depth perception, binocular disparity, 3D image, 2D layout, item distance, area of interest, gaze distribution

## Abstract

Precise perception of three-dimensional (3D) images is crucial for a rewarding experience when using novel displays. However, the capability of the human visual system to perceive binocular disparities varies across the visual field meaning that depth perception might be affected by the two-dimensional (2D) layout of items on the screen. Nevertheless, potential difficulties in perceiving 3D images during free viewing have received only a little attention so far, limiting opportunities to enhance visual effectiveness of information presentation. The aim of this study was to elucidate how the 2D layout of items in 3D images impacts visual search and distribution of maintaining attention based on the analysis of the viewer’s gaze. Participants were searching for a target which was projected one plane closer to the viewer compared to distractors on a multi-plane display. The 2D layout of items was manipulated by changing the item distance from the center of the display plane from 2° to 8°. As a result, the targets were identified correctly when the items were displayed close to the center of the display plane, however, the number of errors grew with an increase in distance. Moreover, correct responses were given more often when subjects paid more attention to targets compared to other items on the screen. However, a more balanced distribution of attention over time across all items was characteristic of the incorrectly completed trials. Thus, our results suggest that items should be displayed close to each other in a 2D layout to facilitate precise perception of 3D images and considering distribution of attention maintenance based on eye-tracking might be useful in the objective assessment of user experience for novel displays.

## Introduction

As the demand for high-quality three-dimensional (3D) images keeps growing, various head-mounted and front-view displays are developed and offered on the market (12; 37). Despite the common interest in the increased implementation of 3D images in different professional areas, two-dimensional (2D) images remain useful and in demand. It is expected that 3D displays will someday substitute conventional flat-panel monitors as there will be no need for keeping old technologies once novel displays can display both 3D and 2D images.

Thus, in the field of user experience, one of the key interests lies in the objective assessment of whether 3D and 2D images are quickly and accurately discerned when displayed on the same display.

The human ability to discern 3D images relies on the processing of depth cues (26). From all cues, the relative binocular disparity is considered a prerequisite for accurate depth judgments at close viewing distances (1Howard & Rogers, 2012; 33). It has been shown both in neurophysiological and behavioral studies that the information about binocular disparity is available early in visual processing (2; 3; 23; Pegna et al., 2018). Moreover, it contributes to the saliency of the visual scene and spatial deployment of attentional resources (10; 23; 28).

Visual information is captured through a series of fixations interrupted by saccadic eye movements (7). Most fixations land on the parts of images that attract visual attention (18; 22; 32). Thus, information about eye movements can be used to measure the attention that individuals have paid to the visual scene (8; 15; Vries et al., 2017). This is one of the reasons why eye-tracking has become increasingly popular in the assessment of 3D presentation of information on the cognitive processes of viewers (5; 15; 20; 29) and in the development of meaningful gaze-based interaction methods for 3D user interfaces (1; Hadnett et al., 2019; Kang et al., 2020).

Eye-tracking studies have complemented the findings of psychophysical studies by showing that eye movements can be disparity-driven (13; 22; 21). However, it has been also observed that disparity-based image saliency does not always dictate the spatial deployment of fixations. Specifically, if the visual task is difficult, 3D images are viewed in a systematic way (29).

Previous research manipulating the target-distractor similarity in 2D images (4) has shown that the viewers’ gaze is more focused on the target when target-distractor similarity is low in visual search. However, with an increase in target-distractor similarity, a similar amount of time can be devoted to viewing each item of the search display.

It is important to keep in mind that the interpretation of image depth depends on the capability of the human visual system to perceive binocular disparities across the visual field. There are certain limitations in discrimination of binocular disparities when comparing the foveal and peripheral visual fields which contribute to the ability of processing information about image depth. Specifically, as retinal eccentricity increases, the efficiency of depth perception decreases. By experimentally determining the magnitude of the minimum depth difference that a person can distinguish, it was shown that sensitivity gradually decreases with increasing retinal eccentricity up to 5-6°, while a sharp decrease in sensitivity is observed at larger eccentricities (6; 24; 31; 36). Moreover, a general decrease in attentional capacity is observed as stimulus eccentricity increases, irrespective of stimulus magnification (34). Thus, these perceptual limitations might affect the ability to discern image depth depending on the 2D layout of items in the 3D visual scene during free viewing, however, the effect of target location on 3D visual search has been most studied in the context of relative depth (Z dimension) (e.g., 10; 9).

The aim of the current study was to elucidate how the 2D layout of items in 3D images impacts visual search and distribution of maintaining attention among targets and distractors by manipulating the item distance – the distance of items from the center of the display plane in X and Y dimensions. It was expected that if a person discerns a 3D effect, they will pay more attention to the target compared to distractors. Furthermore, it was hypothesized that as the item distance increases, it will become more difficult to discern the target. Lastly, it was proposed that if a person does not discern the disparity-defined target correctly, attention will be distributed across 3D image items in a similar way as across 2D image items.

## Method

### Participants

Twelve adults (2 males, 10 females; convenience sample based on the heuristics justification (17)) with a mean age of 22 years (range: 21-25 years) voluntarily took part in the study. To assure sufficient visual discrimination abilities, visual functions were assessed before the experiment. The participants’ inclusion criteria were the following: normal or corrected-to-normal (with contact lenses) visual acuity (1.0 or better, decimal units), stereoscopic acuity of 40 arcsec or better (assessed using a Titmus stereotest, Stereo Optical Co., Chicago, IL). All participants were unaware of the specific purpose of the study. The study was approved by the Ethics Committee of the University of Latvia and was conducted in accordance with the Declaration of Helsinki.

### Apparatus

Images were presented on a multi-plane display (LightSpace Technologies, model: x1907, 19” diagonal). The display contains twenty image depth planes which are transient liquid-crystal based light diffusers acting as the temporary image receiving screens when synchronously coupled to an image projection unit. In operation, diffusers are switched between the transparent and light diffusing state, ensuring an overall image refresh rate of 60 Hz. The resolution of the displays is 1024 × 768 pixels per image depth plane. The distance between sequential image depth planes is 5.04 mm. All displayed images contained bright elements (21.0 cd/m2 luminance) presented on a dark background (0.6 cd/m2 luminance, measured with the Photo Research spectroradiometer PR-655).

Binocular eye movements were recorded using an EyeLink 1000 Plus (SR Research Ltd.) eye-tracker with a Desktop Mount operating at a sampling rate of 500 Hz. The eye-tracker equipment included a chin and forehead rest to prevent head movements and ensure that the 60 cm distance between the subject's eyes and the multi-plane display was consistently maintained.

### Procedure and task

First, the procedure and the aims of the task were explained to the participants. Then, the participants provided written informed consent. Next, participants’ visual acuity and stereoscopic acuity were assessed. The visual tasks were conducted in a dimly lit room.

A nine-point binocular eye movement calibration was performed for the eye-tracker at the beginning of each block (i.e., every 40 trials) using a calibration software that was made for the multi-plane display based on the principles of the commercially available software. To maintain a stable fixation, a combination of circle, dot, and cross (35) was used as a fixation stimulus in the software. The size of the fixation stimulus was 0.6°, and the size of the dot and width of the cross line was 0.2°. The fixation stimulus appeared for 1 sec at each position on the screen. The calibration was confirmed if the measurement error resulting from the data validation was less than 1°.

**Figure 1.**
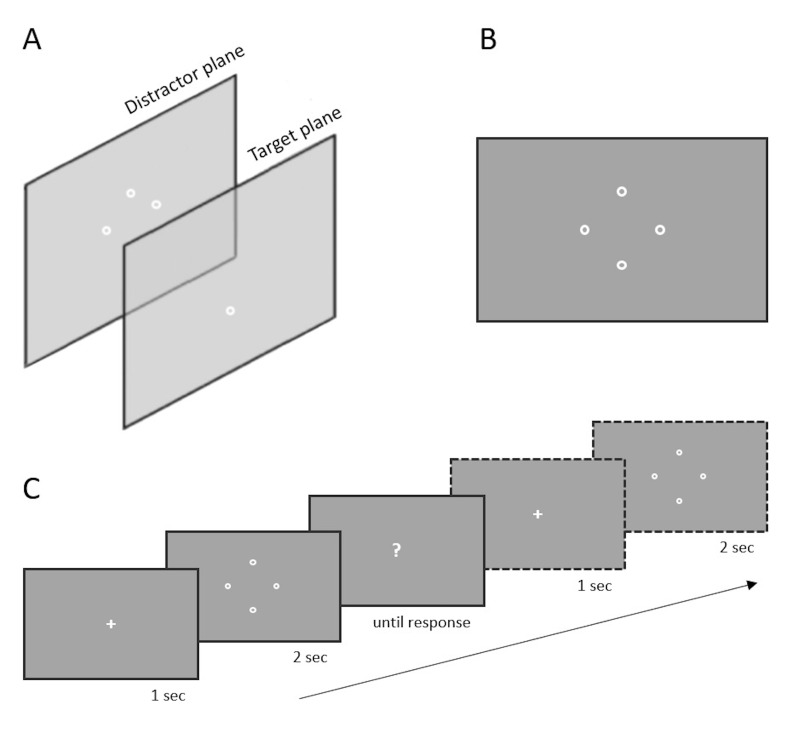
(A) Side view and (B) front view of the item layout. The target was displayed one plane closer to the viewer (Target plane) compared to distractors (Distractor plane) within the optical element of the multi-plane display. (C) The visual task sequence. The fixation cross appeared for one second at the beginning of each trial. Then four circles were shown on the screen. The trial finished with the submission of the subject’s response when the question mark was shown. The illustrations are not to scale.

Each trial started with a fixation cross that was presented in the middle of the third screen plane for 1 sec (61 cm distance from the participant's eyes). Apart from the instruction to fixate the central fixation cross until the circles appeared, no other instructions with regard to eye movements were given. Next, four circles (outer diameter – 0.6°, line width – 0.2°) were displayed for 2 sec. The choice of time limitation was justified by previous research (25). In 3D image (target-present) trials, one circle was shown one image depth plane (5 mm) closer to the viewer in comparison to three others (see Figure 1A, B).

Then the question mark appeared on the screen, and participants reported the closest circle’s relative location within the display by choosing one of four responses (up, right, down, and left). For the response, the arrow keys of a computer keyboard were used. After the response submission, the fixation cross reappeared, and the next trial followed (see Figure 1C). All positions of the closest item were counterbalanced across directions. For the control condition, 2D image (target-absent) trials were added. In 2D image trials, all circles were presented at one depth plane. The subjects responded to 2D images by pushing the space button on the keyboard when the question mark was shown on the screen.

**Figure 2.**
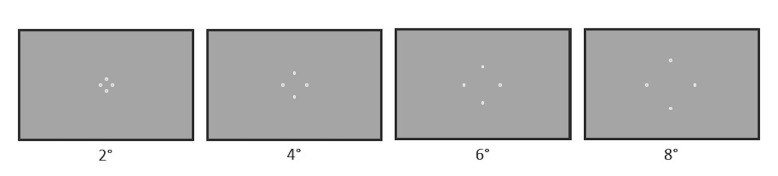
The layout of circles depending on the item distance from the center of the display plane. The illustration is not to scale.

The 2D layout of items was manipulated by changing the item distance from the center of the display plane (see Figure 2). Trials were blocked according to the item distance. The circles were presented at four item distances which were 2°, 4°, 6°, and 8°. The sequence of item distances was randomized among subjects. Each block consisted of 20 3D image trials and 20 2D image trials shown in a pseudo-randomized order. The total number of trials was 160 per subject.

### Data analysis

To assess the ability to discern 3D images depending on the 2D layout of items on the multi-plane display, the data of true-false of the response (in the form of 1 or 0) were recorded. The correct response rate was calculated by dividing the number of correctly completed trials by the sum of all trials for each item distance.

The data analysis was performed using R Statistical Software version 4.0.5 (Foundation for Statistical Computing, Vienna, Austria). Repeated measures ANOVAs were performed to analyze the effects of within-subjects independent variables (item distance and image type) on the correct response rates. For the post hoc comparisons, pairwise t-tests with Bonferroni correction were used. Statistical tests were performed at ɑ = 0.05 significance level.

The results of the binocular eye movement measurements were obtained using the Data Viewer program in the form of X and Y coordinates. Then, mean gaze coordinates (in pixels) considering both eyes were extracted. The coordinates coinciding with blinks, including the coordinates 10 ms before and after blinking, were removed from the resulting data.

As people tend to spend more time looking at parts of the visual scene that attract more attention in comparison to others, the Area-of-Interest (AOI) methodology is useful in the analysis of user experience (14; Rim et al., 2021). We examined the distribution of attention maintenance which is the characteristic of an AOI analysis. To measure the distribution of attention maintenance total dwell time was calculated. Total dwell time is the time that gaze remains in a particular AOI throughout the visual task (16). An AOI with a higher mean total dwell time is assumed to maintain attention longer than other AOIs. The limited-radius Voronoi tessellation method (14) was used to construct AOIs. Namely, the AOIs enclosed the item shapes and included the area within 1° of visual angle around each of four items displayed higher, to the right, lower, and to the left from the center of the depth plane, hereinafter referred to as the positions – up, right, down, and left.

## Results

### Correct response rate

**Figure 3.**
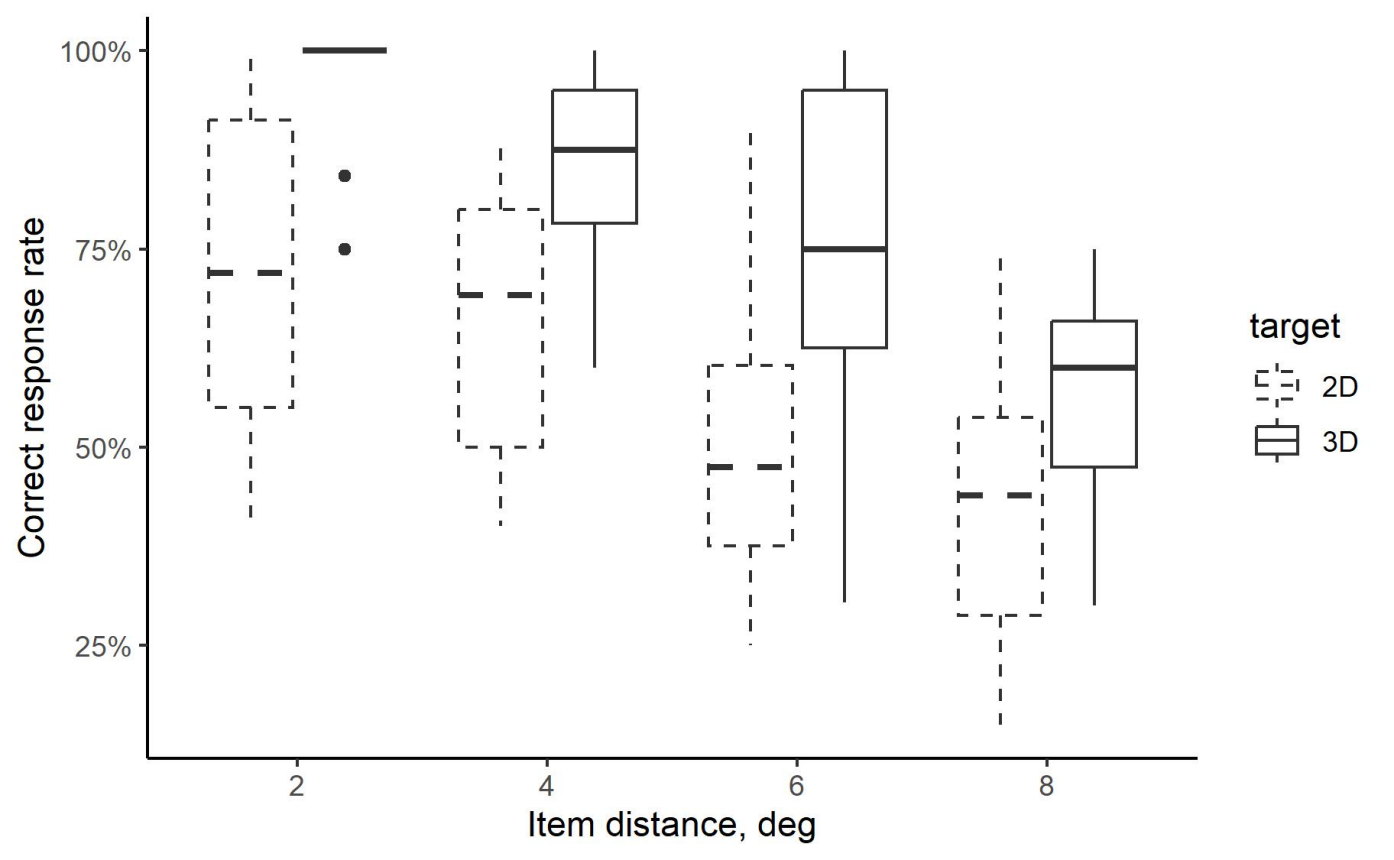
Mean correct response rates for all subjects when responding to 3D images (target-present trials) and 2D images (target-absent trials) with items presented at four distances from the plane center on the multi-plane display. Box plots present median and interquartile range.

The results showed that targets in most 3D images (target-present trials) were discerned correctly when the items were presented at the smallest item distance, which was reflected in high mean correct response rates, and low variability of data (see Figure 3). However, as the item distance increased, the correct response rates declined, and the variability of data grew.

In comparison to 3D images, the correct response rates for 2D images (target-absent trials) were lower at all item distances. The statistical analysis revealed a significant effect for the type of image (F(1,11) = 15.2, p < .001) and item distance (F(3,33) = 50.0, p < .001), but no interaction between these two factors (F(3,33) = 0.9, p = .447). Post-hoc analysis using pairwise t-test with Bonferroni correction showed that the correct response rates were significantly different when comparing results at every two item distances. For all subjects and item distances, most errors (62%) in 3D image tasks were because of submitting the response that the 3D image was considered a 2D image (i.e., participants pressed the space key).

### Total dwell time

**Figure 4.**
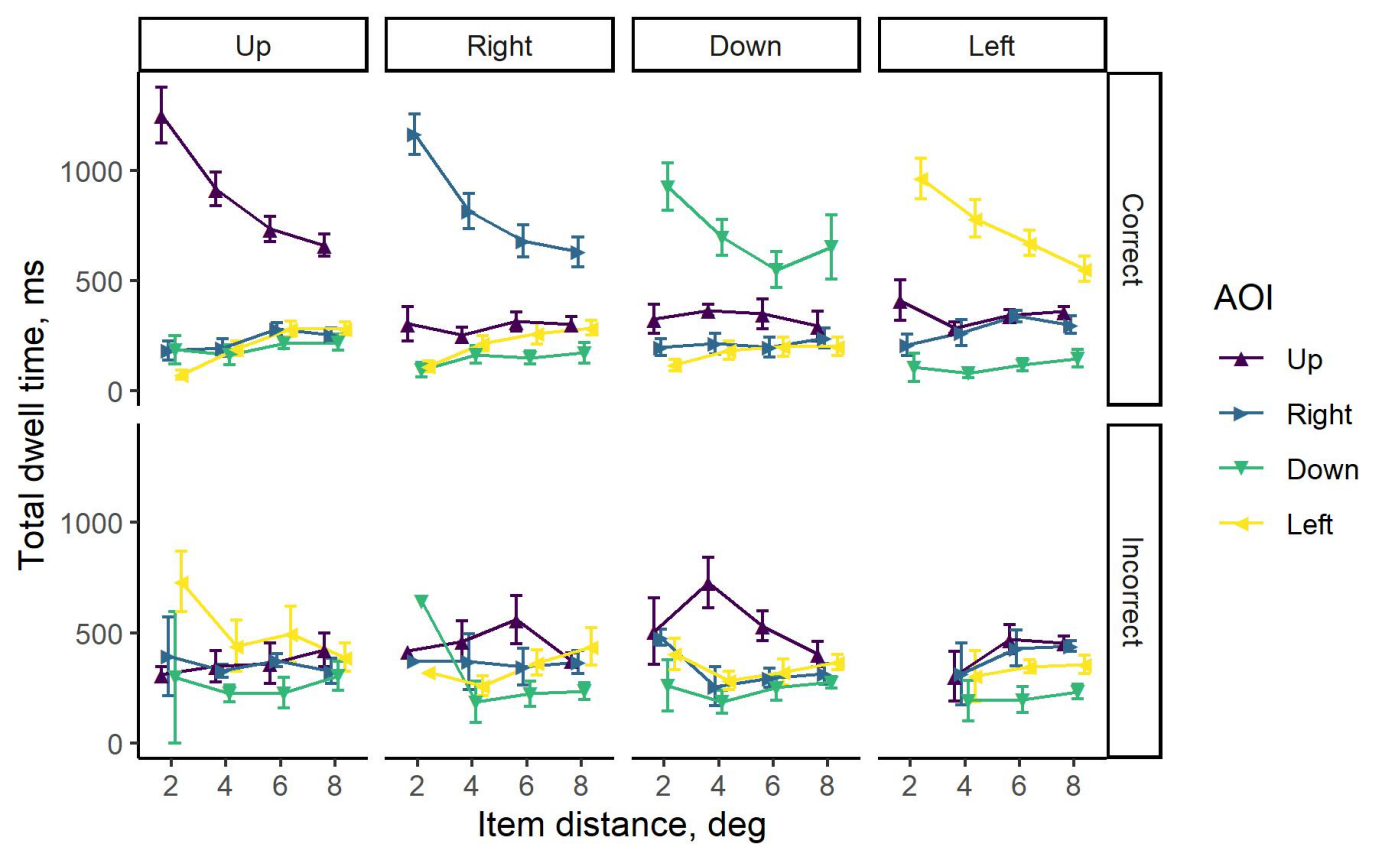
Mean total dwell times for all subjects when responding to 3D images (target-present trials) with items presented at four positions (up, right, down, and left) and four distances from the plane center (2°, 4°, 6°, and 8°) on the multi-plane display. Each column corresponds to a different position of the target. The bars represent the group standard errors. Note that no incorrect responses were submitted when the target was presented to the left from the center of the display plane at 2° distance.

Mean total dwell times for 3D image (target-present) trials with correctly identified targets are plotted in the upper panel of Figure 4, and those with incorrect responses in the lower panel. The results are summarized separately for each target position (up, right, down, and left) and presented in columns. For all target locations within the search display, it can be clearly seen that more time was spent looking at the targets in comparison to distractors when the task was completed correctly. The corresponding difference in total dwell times was the most pronounced for the items located at the smallest distance. Despite the reduction of this difference with an increase in distance, the advantage was still observed. However, no pronounced differences in total dwell times for four AOIs were seen when the wrong responses were given.

**Figure 5.**
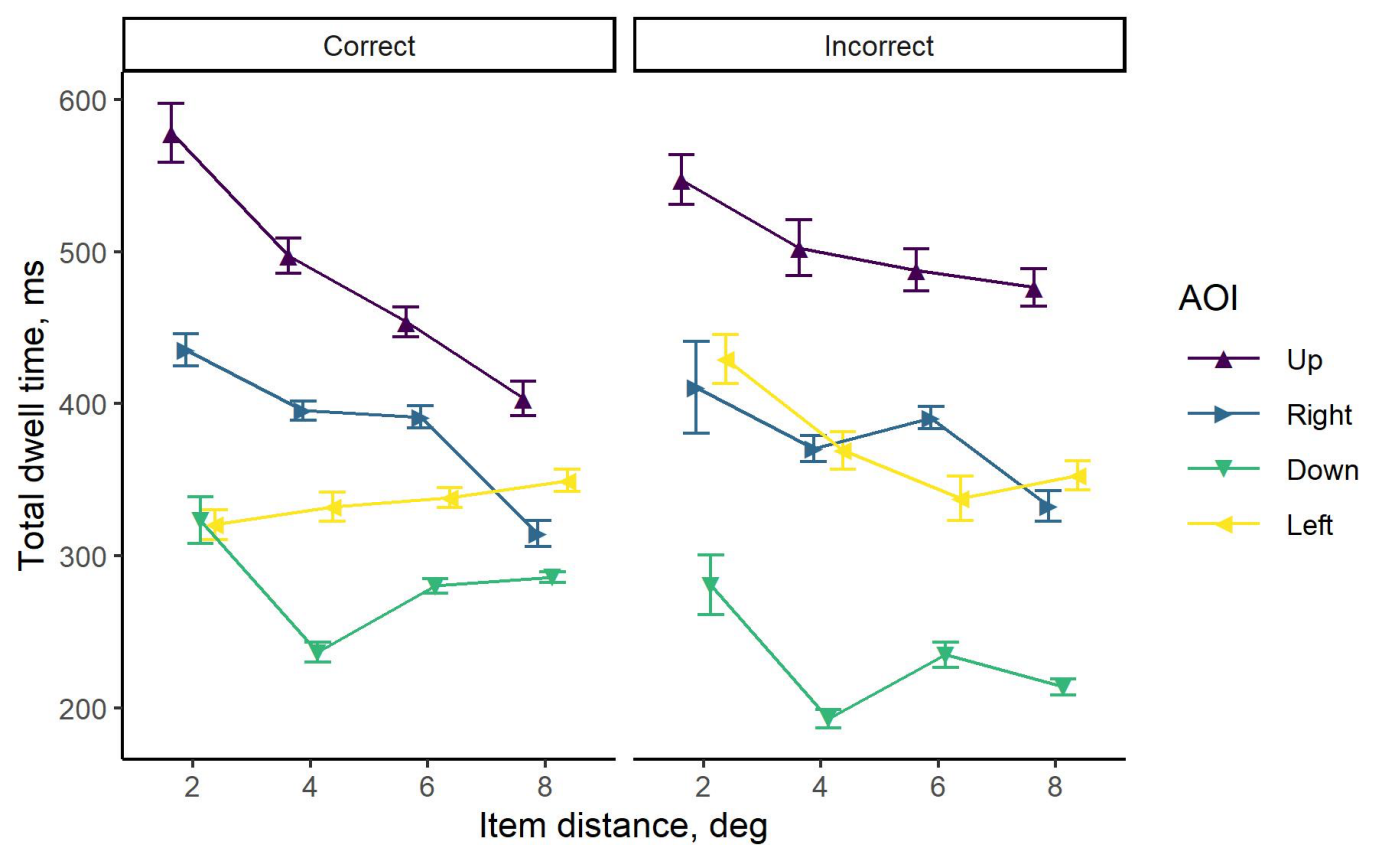
Mean total dwell times for all subjects when responding to 2D images (target-absent trials) with items presented at four positions (up, right, down, and left) and four distances from the plane center (2°, 4°, 6°, and 8°) on the multi-plane display. The bars represent the group standard errors.

As for 2D images (target-absent trials), group means for total dwell times are summarized in Figure 5. A similar pattern is observed when comparing total dwell times across tasks with correct and incorrect responses. Namely, the longest dwell times were observed for AOIs containing the upper item at all item distances, and the shortest dwell times for AOIs containing the lower item.

## Discussion

The user experience of image depth is commonly assessed from the perspective of the depth component (Z dimension). However, the 2D layout of items (X and Y dimensions) may play an important role as the “readiness” of the visual system to perceive depth based on relative binocular disparities varies across the visual field. In the present study, we investigated this by comparing how performance and distribution of attention maintenance across AOIs differ in images shown on the multi-plane display depending on the distance of items from the plane center. The results demonstrate that it becomes more difficult to discern the 3D effect with an increase in item distance. Moreover, at all item distances, paying attention for a longer time to the target compared to distractors is a characteristic of correctly completed tasks.

Depth perception plays an important role in visual search when it comes to finding objects in space or information in 3D images. The multi-plane structure of the display’s optical element allows displaying both 3D and 2D images making it possible to explore how performance and gaze distribution change depending on the layout of items. By showing items on successive image depth planes, it has been elucidated that the highest search performance is achieved when the items are displayed close to each other in the horizontal and vertical meridians. Namely, subjects could correctly detect the closest item which was displayed 5 mm closer to the viewer. This demonstrates that they experienced no difficulties in understanding the layout of items presented in the optical element of the multi-plane display. However, performance deteriorated with an increase in item distance. Based on the analysis of gaze distributions across AOIs and the findings of our previous research (25), it can be suggested that the viewers had sufficient time to examine each item with central vision and pay direct attention. Thus, a decrease in correct response rate with an increase in item distance might indicate that information processing from the central visual field was not sufficient to allow classification of an item as a target or distractor when assessing the relative depth of items in the image with a limited number of depth cues.

Small separation (5 mm) of planes in the optical element of the multi-plane display viewed at the distance of 60 cm resulted in approximately 186” relative disparity if we assume that the mean interpupillary distance is 0.065 m (Howard & Rogers, 2012). It is larger than the stereo threshold in the central vision in participants, thus, unsurprisingly, the search results were at the top when the items were located close to the center of the display plane. Nevertheless, it is known that the stereo threshold increases in the direction from the fovea to periphery (31). As the distance between items grew with an increase in item distance, it could be more difficult to discern the target because of lower retinal sensitivity.

Previously, the effect of item distance was explored for 2D images (Carrasco et al., 1995; Scialfa et al., 1998; Wolfe et al., 1998). It was shown that performance may depend on the target location in the visual scene in relation to the gaze fixation before and during the visual search. Namely, the closest to fixation items can be more salient in comparison to others (Wolfe et al., 1998), and thus the physical properties of the image in the parafoveal and peripheral visual field dictate the deployment of attention and programming of eye movements in visual search (Carrasco et al., 1995; Wolfe et al., 1998; Wolfe, 2021).

Our study complements these findings on item distance and contribute to existing knowledge by adding that search results can be associated not only with item distance but also with the property that defines the target. Namely, a 100% correct response rate is expected in search tasks where one should find a red circle among green circles if the subject does not have any color vision anomalies and search time is not limited. However, if it is not possible to decide whether the viewed item is a target or not based on the information processing from the central visual field, more errors can be made with an increase in the distance between items. Practically, it means that there is a necessity to display items close to each other in X and Y dimensions to facilitate disparity-driven depth judgments. Thus, it would be helpful to provide the opportunity to zoom in and out on images allowing to change the distance between items (parts of images) and facilitate depth judgements.

As expected, viewers paid more attention to the target compared to distractors when the targets in 3D images were discerned correctly. However, the distribution of attention maintenance was more balanced across all four AOIs if the target could not be identified correctly. That possibly means that attention was devoted to each item without any pronounced preference when the viewer experienced difficulties in finding the target. As a result, the accumulated uncertainty in decision making could lead to a larger number of errors. A similar viewing strategy was observed in the study where task difficulty was manipulated by changing the target-distractor similarity (4).

We hypothesized that attention would be distributed across items in 3D images in a similar way as 2D images if the response about the closest item was incorrect, however, the analysis of total dwell times for AOIs in the case of 2D images showed a different interesting pattern. Namely, the viewers spent more time looking at the items which were displayed higher and to the right from the center of the display plane in comparison to other items. This asymmetry could be associated with the dominance of top-down processes in visual search in the absence of the target. Specifically, it has been shown that images can be scanned in a specific way – from top to the bottom, possibly because of the dominance of the top-down strategy in difficult visual search (Hwang et al., 2009; 29). Moreover, more attention is paid to the items which are displayed in the upper part of the display if the target is not found at once (Guan & Cutrell, 2007), and the search results can be superior (19; 30). Even though the most errors were made because 3D images were considered 2D images, our findings suggest that visual search strategies differ in target-present and target-absent trials. We speculate that the presence of the disparity-defined target prompted subjects to pay attention to each item and struggling to choose the closest item led to the selection of an incorrect response.

In conclusion, this study has shown that the 2D layout of items in 3D images is important to consider because the perception of image depth is strongest when items are displayed close to each other. Moreover, analyzing the distribution of attention maintenance across AOIs can be useful in the objective assessment of user experience for 3D image displays.

### Ethics and Conflict of Interest

The author(s) declare(s) that the contents of the article are in agreement with the ethics described in http://biblio.unibe.ch/portale/elibrary/BOP/jemr/ethics.html and that there is no conflict of interest regarding the publication of this paper.

### Acknowledgements

This work was funded by the Latvian Council of Science (project No. lzp-2021/1-0399). The authors thank Rendijs Smukulis (LightSpace Technologies) for making the eye-tracking software for the multi-plane display, and Kurt Debono (SR Research) and Aiga Svede (University of Latvia) for the consultation on the specifics of eye tracker calibration.
